# Cigarette smoking and mammographic breast density in post-menopausal women from the EPIC Florence cohort

**DOI:** 10.3389/fonc.2024.1335645

**Published:** 2024-03-07

**Authors:** Benedetta Bendinelli, Saverio Caini, Melania Assedi, Ilaria Ermini, Elisa Pastore, Luigi Facchini, Maria Antonietta Gilio, Giacomo Duroni, Miriam Fontana, Andrea Querci, Daniela Ambrogetti, Calogero Saieva, Giovanna Masala

**Affiliations:** ^1^ Clinical Epidemiology Unit, Institute for Cancer Research, Prevention and Clinical Network (ISPRO), Florence, Italy; ^2^ Cancer Risk Factors and Lifestyle Epidemiology Unit, Institute for Cancer Research, Prevention and Clinical Network (ISPRO), Florence, Italy; ^3^ Breast Cancer Screening Branch, Institute for Cancer Research, Prevention and Clinical Network (ISPRO), Florence, Italy

**Keywords:** cigarette smoking, mammographic breast density, post-menopausal women, breast cancer, lifestyle

## Abstract

**Introduction:**

Cigarette smoking has been recognized as a risk factor for breast cancer (BC) also if the biological mechanism remains poorly understood. High mammographic breast density (MBD) is associated with BC risk and many BC risk factors, such as genetic, anthropometric, reproductive and lifestyle factors and age, are also able to modulate MBD. The aim of the present study was to prospectively explore, in post-menopausal women, the association between smoking habits and MBD, assessed using an automated software, considering duration and intensity of smoking.

**Methods:**

The analysis was carried out in 3,774 women enrolled in the European Prospective Investigation into Cancer and Nutrition (EPIC) Florence cohort in 1993-98, participating in the 2004-06 follow up (FU) and with at least one full-field digital mammography (FFDM) performed after FU. For each woman, detailed information on smoking habits, anthropometry, lifestyle and reproductive history was collected at enrollment and at FU. Smoking information at baseline and at FU was integrated. The fully automated Volpara™ software was used to obtain total breast volume (cm^3^), absolute breast dense volume (DV, cm^3^) and volumetric percent density (VPD, %) from the first available FFDM (average 5.3 years from FU). Multivariable linear regression models were applied to evaluate the associations between smoking habits and VPD or DV.

**Results:**

An inverse association between smoking exposure and VPD emerged (Diff% -7.96%, p <0.0001 for current smokers and -3.92%, p 0.01 for former smokers, compared with non-smokers). An inverse dose-response relationship with number of cigarettes/day, years of smoking duration and lifetime smoking exposure (pack-years) and a direct association with time since smoking cessation among former smokers emerged. Similar associations, with an attenuated effect, emerged when DV was considered as the outcome variable.

**Discussion:**

This longitudinal study confirms the inverse association between active smoking, a known risk factor for BC, and MBD among post-menopausal women. The inclusion of smoking habits in the existing BC risk prediction models could be evaluated in future studies.

## Introduction

Breast cancer (BC) is the most commonly diagnosed cancer and the leading cause of cancer death among women worldwide and in all European countries (1, 2). BC risk factors include environmental and genetic factors. Some of these risk factors, such as women’s age, family history, menstrual and reproductive history, are non-modifiable (3). Other risk factors are modifiable and related to lifestyle characteristics including lack of physical activity and alcohol intake (4). In the 2012 monograph 100E on Personal Habits and Indoor Combustions, the International Agency for Research on Cancer (IARC) concluded that there was a direct association between tobacco smoking and female BC risk (5). Subsequently, a recent review and meta-analysis conducted on the basis of 169 studies, provided further evidence of the causal role of tobacco smoke on the risk of BC, with an increased risk of 7%, 8% and 9% respectively in current smokers, former smokers and ever smokers, compared to non-smokers (6).

Mammographic breast density (MBD) refers to the proportion of radiologically dense fibroglandular tissue over the total breast, and is usually expressed as percent density. High MBD is an independent risk factor for BC, with consisting evidence that has steadily accumulated in recent years. Many studies have shown that women with high MBD have a two-to-six fold increased risk of BC compared to women with low MBD (7–11).

MBD can be modulated by known BC risk factors (12). Parity, early age at first birth, consumption of vegetables, intake of antioxidants, and increased PA are reported to be inversely associated with both MBD and BC risk. Hormone replacement therapy and intakes of protein, saturated fat, and alcohol are reported to be directly associated with both MBD and BC risk (13–23). On the other hand, age and body mass index (BMI) are inversely associated with MBD but positively associated with BC risk (12).

Most of the previous epidemiological studies on the relationship between smoking status and mammographic density had a cross-sectional design and found a lower mammographic density among current smokers as compared to non-smokers, both among menopausal and non-menopausal women (24–27). Some studies also evaluated the effect of smoking duration or intensity (16, 28–30). As regards the methods for MBD assessment, these previous studies mostly relied on non-quantitative methods (BI-RADS, Boyd’s scale) and more recently on computer-assisted programs on digitized images obtaining dense and non-dense area measurements of the breast (Cumulus, Madena). Over the last decade, full-field digital mammography (FFDM) has progressively replaced film-screen mammography in screening programs and the development of automated softwares for volumetric MBD assessment allowed to obtain volumetric MBD measures.

The aim of the present study was to explore, in a series of post-menopausal women participating in the European Prospective Investigation into Cancer and Nutrition (EPIC) Florence cohort, the association between smoking habits and MBD assessed using an automated programs and considering in detail the duration and intensity of smoking. The prospective approach of this study made it possible to evaluate the effect of cigarette smoking on MBD after some years from the exposure, also considering the effect of changes in smoking habits.

## Materials and methods

### Study population

Between 1993 and 1998, 10,083 clinically healthy women aged 35–64 years residing in the Florence area (Tuscany, Central Italy) were recruited in the EPIC Florence cohort. All study participants signed an informed consent and gave permission to use the data collected during the study. The study was approved by the local Ethics Committee “Azienda Sanitaria Firenze”. All procedures were in accordance with the ethical standards of the institutional and national research committee and with the 1964 Helsinki declaration and its later amendments or comparable ethical standards.

Body weight (kg), body height (cm), waist and hip circumferences (cm) were obtained by trained nurses according to an international protocol. Detailed information on reproductive history, smoking and alcohol drinking history, educational level (none/primary school, secondary/professional school, high school, university), physical activity habits, medical history and hormone replacement therapy (HRT) use was collected through a standardized lifestyle questionnaire (LSQ). Dietary information was obtained through a validated Food Frequency Questionnaire (FFQ). Standardized follow-up procedures have been periodically implemented for the ascertainment of vital status and the identification of cancer cases diagnosed after enrollment (31).

Information on lifestyle, medical history, reproductive history and anthropometric measures was updated in 2004–2005, after a 9.4 year average follow-up, through a specific questionnaire (32).

In 2019, in the frame of the FEDRA (Florence-EPIC Digital mammographic density and breast cancer Risk Assessment) study, an assessment of the mammographic examinations history of the EPIC female participants was performed through a linkage with the mammographic archives of the local population-based mammographic screening program (performed at ISPRO, in charge of the mammographic screening in the area). For the available FFDMs, quantitative MBD measures were obtained. The FEDRA study was approved by the Ethics Committee of “Area Vasta Centro” of Tuscany Region and participants signed a specific consent form (33).

**Figure 1:**
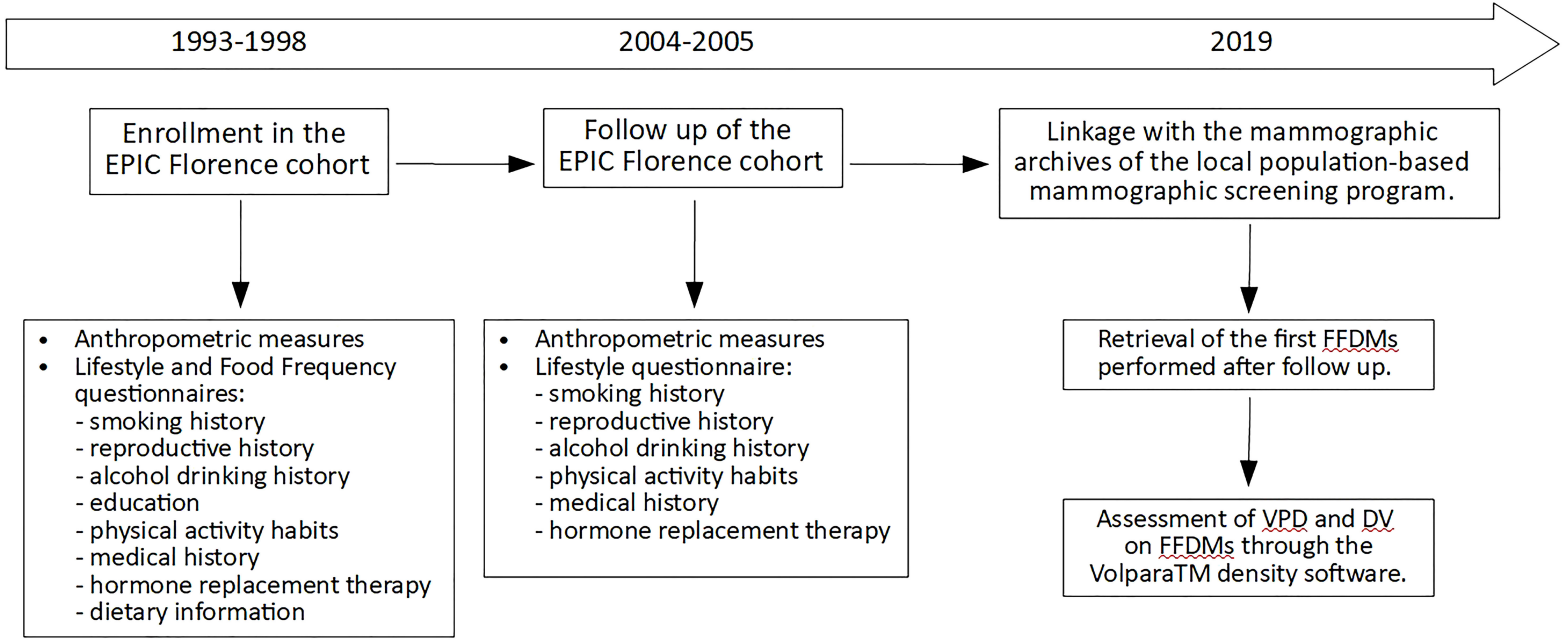
Timeline diagram of the prospective study on the 3,774 menopausal women from the EPIC Florence cohort. FFDM, full-field digital mammography; VPD, volumetric percent density; DV, dense volume.

The present analysis was focused on 3,774 women, previously enrolled in the Florence-EPIC study, for which 2004–2005 follow-up data were available and with at least a FFDM performed after menopause and after 2004–2005 follow-up (**Figure 1**).

### Breast density measures

The fully automated Volpara™ density software (version 3.1, Matakina Technology, Wellington, New Zealand) was used to obtain total breast volume (cm^3^), absolute breast dense volume (DV, cm^3^) and volumetric percent density (VPD, %), from raw (“for processing”) FFDMs data of the retrieved mammograms. The technical characteristics of the Volpara system have been already described in details (34). Briefly, the algorithm computes the thickness of dense tissue at each pixel using the X-ray attenuation of an entirely fatty region as an internal reference. The thickness values over the whole breast region are integrated to obtain the absolute DV. Total breast volume is obtained by multiplying the breast area by the recorded breast thickness, corrected for the breast edge. VPD was then obtained from the ratio of DV and total breast volume. In this study, we used the average MBD measures obtained from medio-lateral oblique and cranio-caudal views of the right and left breasts on the first FFDM performed after 2004–2005 follow-up.

### Smoking history

The baseline EPIC LSQ and the 2004–2005 follow-up questionnaire included specific sections aimed to assess cigarette smoking history including: smoking status (current, former or never smoker); smoking dose (for current smokers, number of cigarettes smoked per day); age at smoking onset (for current and former smokers); and age at quitting smoking (for former smokers) (31, 32). In the baseline EPIC LSQ volunteers were also asked to recall the number of cigarettes smoked per day at 20, 30, 40, and 50 years of age. Information from both the baseline and the 2004–2005 follow-up questionnaires was integrated and participants’ smoking status at follow-up was assessed.

### Statistical analysis

Main characteristics of the 3,774 study women (number, means and standard deviations for continuous variables, distribution and percentages for categorical variables) were calculated. Body mass index (BMI) was calculated as weight (kg) over squared height (m^2^).

For each participant, we computed the birth index combining the age at every birth and their number. Birth index was 0 for nulliparous. A higher birth index indicates a higher number of births occurring at earlier ages (35).

We used educational level for the calculation of the relative index of inequality (RII), an indicator of socio-economic status that overcomes possible differences in the proportion of subjects in the educational levels across 10-years birth groups. A low RII represented a high level of education (36, 37).

The daily alcohol intake was expressed as number of alcoholic units consumed daily (1 alcoholic units = 12 g, average alcohol amount in 1 drink).

Pack-years were computed as a cumulative index of lifetime consumption of cigarettes (the sum of the number of cigarettes smoked per day in each year of life up until 2004-2005 follow-up, divided by 20, the number of cigarettes in a pack) (38).

Multivariable linear regression models were applied to evaluate the associations between smoking habits and VPD and between smoking habits and DV. Smoking exposure was analyzed according to: smoking status categories (never smokers, former smokers, current smokers); smoking intensity categories (never smokers, current smokers of 1–5, 6–10, 11-15, >15 cigarettes/day based on quartiles); age at smoking onset categories (never smokers, smokers with onset at age >20, 18–20, ≤17, based on tertiles); smoking duration categories (never smokers, smokers with duration of 1–10, 10–20, 20-30, >30 years); lifetime smoking exposure categories (never smokers, smokers of 0-6, 6-15, 15-23, >23 pack-years, according to quartiles); time since cessation categories (current smokers, former smokers from 0-10, 10-20, >20 years). Categorical variables were treated as dummy variables in regression models, with never smokers as reference category, except for time since cessation analysis where current smokers was the reference category. The linearity of trends across categories was tested by treating categories as a continuous variables. All models were adjusted for age at menarche (years, continuous), age at menopause (years, continuous), birth index (continuous), BMI at follow-up (kg/m^2^, continuous), waist circumference at follow-up (cm, continuous), RII (continuous), HRT at follow-up (yes/no), daily alcoholic units at follow-up (continuous), age at mammographic examination (years, continuous), and mammographic breast thickness (mm, continuous).

Specific models with the same adjustments were implemented in order to evaluate the effect, on VPD and DV, of any changes in smoking habits between baseline and follow-up. The effect of smoking onset or smoking cessation after baseline, and the effect of the variation in number of cigarettes smoked per day between baseline and follow-up among current smokers (model further adjusted for number of cigarette/day at baseline) were investigated.

In all models, VPD and DV were log-transformed in order to normalize the distribution. Beta coefficients and 95% confidence intervals (CI) were then back transformed and presented as percent change in VPD or DV (Diff% = (expβ-1)×100) with respect to the reference category.

Estimates of the single individual predicted values of VPD and DV were obtained as post-estimation analysis and used to calculate the adjusted means and 95% CIs for VPD and DV according to smoking habits categories.

As sensitivity analyses, quadratic b-splines were generated with knots at the mid values of categories of smoking intensity (number of cigarettes/day) and lifetime smoking exposure (pack-years), respectively. Regression models were then performed and used to predict fitted values for log-transformed VPD. Relative B- spline curves were then implemented.

The analyses were performed using Stata 14.1 statistical software (StataCorp LLC, College Station, TX, USA).

## Results

Main characteristics of the 3,774 study women, overall and according to smoking status at follow-up, are reported in **Table 1**. Mean age at EPIC enrollment was 49.8 years, mean age at follow up was 59.6 years and mean age at first FFDM after follow up was 64.9 years with mean 5.3 years between follow-up and FFDM. At enrollment, current smokers were significantly younger compared to never smokers, had a lower parity, a lower BMI, a higher educational level and a higher alcohol intake.

**Table 1:** Characteristics of the 3,774 menopausal study women from the EPIC Florence cohort by smoking status.

Characteristics	Total women n=3,774	Smoking status at follow up			p value
		Never smokers n=1,798	Former smokers n=1,272	Current smokers n=704	
**Age at EPIC enrollment (years)**	49.8 (6.5)	50.7 (6.5)	49.0 (6.3)	48.6 (6.7)	<0.0001 ^a^
**Age at follow-up (years)**	59.6 (6.3)	60.6 (6.2)	59.0 (6.1)	58.5 (6.5)	<0.0001 ^a^
**Age at FFDM (years)**	64.9 (5.9)	65.7 (5.8)	64.4 (5.7)	63.9 (6.2)	<0.0001 ^a^
**Years from follow-up to FFDM**	5.3 (2.7)	5.2 (2.7)	5.4 (2.7)	5.4 (2.7)	0.13 ^a^
Mammographic features					
Volumetric percent density (%)	7.6 (4.9)	7.7 (4.9)	7.4 (4.7)	7.8 (5.0)	0.66 ^a^
Dense volume (cm^3^)	46.4 (24.3)	46.8 (25.3)	46.8 (23.6)	44.7 (23.0)	0.06 ^a^
**Age at menarche (years)**	12.4 (1.4)	12.4 (1.4)	12.3 (1.4)	12.3 (1.4)	0.03 ^a^
**Age at menopause (years)**	50.1 (4.1)	50.3 (4.1)	50.2 (4.1)	49.5 (4.2)	<0.0001 ^a^
Parity (n)					
Nulliparous	543 (14.4)	225 (12.5)	191 (15.0)	127 (18.0)	
One	1,117 (29.6)	513 (28.5)	395 (31.1)	209 (29.7)	
Two	1,681 (44.5)	825 (45.9)	559 (44.0)	297 (42.2)	
Three or more	433 (11.5)	235 (13.1)	127 (9.9)	71 (10.1)	0.02 ^b^
**Age at first childbirth among parous women (years)**	26.7 (4.4)	26.5 (4.2)	26.9 (4.5)	26.7 (4.5)	0.49 ^a^
**HRT at follow-up (n)**	288 (7.6)	137 (7.6)	93 (7.3)	58 (8.2)	0.61 ^b^
Educational level at EPIC enrollment (n)					
None/e primary school	837 (22.2)	503 (28.0)	201 (15.8)	133 (18.9)	
Secondary/professional school	1,206 (31.9)	585 (32.5)	403 (31.7)	218 (31.0)	
High school	1,018 (27.0)	419 (23.3)	381 (29.9)	218 (31.0)	
University	713 (18.9)	291 (16.2)	287 (22.6)	135 (19.2)	<0.0001 ^b^
**BMI at follow-up (kg/m^2^)**	25.7 (4.21)	25.7 (4.2)	26.0 (4.3)	25.1 (4.1)	0.004 ^a^
**Waist circumference at follow-up (cm)**	84.9 (11.0)	84.6 (10.6)	85.6 (11.3)	84.2 (11.2)	0.46 ^a^
**Alcohol drinkers at follow-up (n)**	3,284 (87.0)	1,524 (84.8)	1,148 (90.3)	612 (86.9)	<0.0001 ^b^
**Alcohol intake at follow-up among drinkers (g/day)**	10.6 (14.5)	8.6 (11.2)	11.2 (15.1)	14.7 (19.2)	<0.0001 ^a^
Smoking status at baseline					
Never smokers	1,817 (48.1)	1,798 (100.0)	11 (0.9)	8 (1.1)	
Former smokers	958 (25.4)	0 (0.0)	910 (71.5)	48 (6.8)	
Current smokers	999 (26.5)	0 (0.0)	351 (27.6)	648 (92.1)	<0.0001 ^b^

Data are presented as means (SD) for continuous variables or number (%) for categorical variables.

FFDM, full filled digital mammogram; HRT, hormonal replacement therapy; BMI, body mass index.

^a^p value from t test for current smokers versus never smokers ^b^p value from Chi-squared test.

The results of the multivariable linear regression for VPD and DV are presented in **Tables 2**, **3** together with the respective adjusted means and 95% CI. Both the current and former smokers at follow-up had a significantly lower VPD compared to non-smokers (Diff% -7.96%, p <0.0001 and -3.92%, p 0.01, respectively) while the observed inverse association with in DV was not significant. Increasing duration of smoking and increasing number of cigarette smoked per day were inversely associated with both VPD and DV. In particular, women smoking for more than 30 years had a 8.01% reduction in VPD (p<0.0001, p trend <0.0001) and a 4.30% reduction in DV (p 0.02, p trend 0.06) with respect to never smokers. Among current smokers at follow-up, women smoking more than 15 cigarettes per day had a 9.43% reduction in VPD (p 0.002, p trend <0.0001) and a 7.34% reduction in DV (p 0.04, p trend 0.02) in comparison to never smokers. Moreover, a younger age at smoking onset was associated with lower VPD (p trend <0.0001) and lower DV (p trend 0.04). Smokers since age ≤17 had a 7,91% lower VPD (p <0.0001) and a 5.39% lower DV (p 0.01) compared to never smokers. Finally former smokers since 20 years or more experimented a 5.87% increased VPD (p 0.02, p trend 0.01) respect to current smokers.

**Table 2:** Association between smoking habits and volumetric percent mammographic density (VPD, %) among the 3,774 menopausal study women from the EPIC Florence cohort.

	N (%)	Adjusted mean of VPD (95%CI) ^a^	Diff% ^b^	95%CI	p-value
Smoking status at follow-up					
Never smokers	1,798 (47.6)	6.61 (6.49; 6.74)	Ref.		
Former smokers	1,272 (33.7)	6.28 (6.14; 6.43)	-3.92	-6.79; -0.96	0.01
Current smokers	704 (18.7)	6.59 (6.39; 6.80)	-7.96	-11.29; -4.51	<0.0001
Age at smoking onset among current and former smokers (years)					
Never smokers	1,798 (47.6)	6.61 (6.49; 6.74)	Ref.		
>20	589 (15.6)	6.13 (5.92; 6.33)	-2.55	-6.25; 1.30	0.192
18-20	785 (20.8)	6.50 (6.32; 6.70)	-5.76	-9.02; -2.38	0.001
≤17	602 (16.0)	6.51 (6.30; 6.73)	-7.91	-11.51; -4.17	<0.0001
p-trend					<0.0001
Smoking duration among current and former smokers (years) ^c^					
Never smokers	1,798 (47.7)	6.61 (6.49; 6.74)	Ref.		
0-10	298 (7.7)	6.70 (6.40; 7.03)	-3.12	-7.99; 2.00	0.23
11-20	379 (10.1)	6.52 (6.25; 6.80)	-1.96	-6.40; 2.70	0.40
21-30	432 (11.5)	6.23 (5.99; 6.47)	-4.23	-8.34; 0.05	0.05
>30	865 (23.0)	6.32 (6.15; 6.50)	-8.01	-11.12; -4.80	<0.0001
p-trend					<0.0001
Lifetime smoking exposure (pack-years) among current and former smokers					
Never smokers	1,798 (4.6)	6.61 (6.49; 6.74)	Ref.		
0-6	502 (13.3)	6.84 (6.59; 7.09)	-0.20	-4.46: 4.04	0.93
6-15	482 (12.8)	6.34 (6.11; 6.58)	-5.17	-9.05; -1.13	0.01
15-23	640 (17.0)	6.25 (6.05; 6.45)	-5.48	-8.99; -1.83	0.004
>23	352 (9.3)	6.11 (5.85; 6.38)	-12.38	-16.49; -8.06	<0.0001
p-trend					<0.0001
Number of cigarettes/day among current smokers					
Never smokers	1,798 (71.9)	6.61 (6.49; 6.74)	Ref.		
1-5	157 (6.3)	7.11 (6.66; 7.58)	-0.45	-7.00; 6.56	0.90
6-10	209 (8.4)	6.45 (6.10; 6.82)	-9.46	-14.74; -3.85	0.001
11-15	147 (5.9)	6.32 (5.91;6.75)	-10.47	-16.51; -4.01	0.002
>15	191 (7.6)	6.56 (6.19; 6.96)	-9.43	-15.02; -3.46	0.002
p-trend					<0.0001
Time since smoking cessation among former smokers (years) ^c^					
Current smokers	704 (35.7)	6.59 (6.39; 6.80)	Ref.		
0-10	298 (15.1)	6.03 (5.75; 6.32)	1.71	-3.93; 7.67	0.56
11-20	428 (21.7)	6.29 (6.04; 6.55)	4.83	-0.34; 10.27	0.07
>20	543 (27.5)	6.41 (6.19; 6.64)	5.87	0.98; 10.99	0.02
p-trend					0.01

a Reported means and 95%CI are back-transformed from log-transformed estimated means from multivariable linear regression models.

b Multivariable linear regression models adjusted for age at menarche, age at menopause, birth index, BMI at follow up, waist circumference at follow up, RII, HRT at follow up, daily alcoholic units at follow up, age at mammographic examination, and mammographic breast thickness. The %Diff represents the percent change of VPD with respect to the reference category.

c Because of some missing data, not all numbers add up to the total.

**Table 3:** Association between smoking habits and breast dense volume (DV, cm^3^) among 3,774 menopausal women from the EPIC Florence cohort.

	N (%)	Adjusted mean of DV (95%CI) ^a^	Diff% ^b^	95%CI	p-value
Smoking status					
Never smokers	1,798 (47.6)	41.6 (40.7; 42.4)	Ref.		
Former smokers	1,272 (33.7)	42.2 (41.2; 43.3)	-0.88	-4.14; 2.48	0.60
Current smokers	704 (18.7)	39.9 (38.6; 41.3)	-3.57	-7.41; 0.42	0.08
Age at smoking onset among current and former smokers (years)					
Never smokers	1,798 (47.6)	41.6 (40.7; 42.5)	Ref.		
>20	589 (15.6)	41.9 (40.4; 43.5)	-0.07	-4.25; 4.28	0.973
18-20	785 (20.8)	41.7 (40.4; 43.0)	-0.67	-4.45; 3.26	0.734
≤17	602 (16.0)	40.5 (39.0; 42.0)	-5.39	-9.45; -1.15	0.013
p-trend					0.040
Smoking duration among current and former smokers (years) ^c^					
Never smokers	1,798 (47.7)	41.6 (40.7; 42.4)	Ref.		
0-10	298 (7.7)	42.5 (40.3; 44.8)	0.20	-5.32; 6.05	0.94
11-20	379 (10.1)	42.9 (41.0; 44.9)	0.03	-4.95; 5.28	0.99
21-30	432 (11.5)	43.2 (41.4; 45.1)	0.45	-4.28; 5.41	0.86
>30	865 (23.0)	39.5 (38.3; 40.7)	-4.30	-7.85; -0.62	0.02
p-trend					0.06
Lifetime smoking exposure (pack-years) among current and former smokers					
Never smokers	1,798 (4.6)	41.6 (40.7; 42.4)	Ref.		
0-6	502 (13.3)	42.7 (41.1; 44.5)	1.06	-3.47; 5.80	0.65
6-15	482 (12.8)	42.0 (40.3; 43.7)	-1.02	-5.48; 3.64	0.66
15-23	640 (17.0)	42.0 (40.5; 43.5)	-0.84	-4.89; 3.39	0.69
>23	352 (9.3)	37.7 (35.9; 39.5)	-8.69	-13.40; -3.62	0.001
p-trend					0.01
Number of cigarettes/day among current smokers					
Never smokers	1,798 (71.9)	41.6 (40.7; 42.5)	Ref.		
1-5	157 (6.3)	42.9 (39.9; 46.1)	3.11	-4.39; 11.19	0.43
6-10	209 (8.4)	39.8 (37.4; 42.3)	-3.24	-9.48; 3.43	0.33
11-15	147 (5.9)	39.1 (36.3; 42.1)	-5.30	-12.35; 2.31	0.17
>15	191 (7.6)	38.4 (35.9; 41.0)	-7.34	-13.66; -0.55	0.04
p-trend					0.02
Time since smoking cessation among former smokers (years) ^c^					
Current smokers	704 (35.7)	39.9 (38.6; 41.3)	Ref.		
0-10	298 (15.1)	40.8 (38.8; 43.0)	-0.83	-6.79; 5.50	0.79
11-20	428 (21.7)	43.2 (41.3; 45.0)	4.46	-1.12; 10.36	0.12
>20	543 (27.5)	42.3 (40.7; 43.9)	3.88	-1.32; 9.36	0.15
p-trend					0.07

a Reported means and 95%CI are back-transformed from log-transformed estimated means from multivariable linear regression models.

b Multivariable linear regression models adjusted for age at menarche, age at menopause, birth index, BMI at follow up, waist circumference at follow up, RII, HRT at follow up, daily alcoholic units at follow up, age at mammographic examination, and mammographic breast thickness. The %Diff represents the percent change of DV with respect to the reference category.

c Because of some missing data, not all numbers add up to the total.

As regards the effect of changes in smoking habits between baseline and follow-up, no significant association with VPD emerged among the 56 women (48 former smokers at baseline) who started or re-started smoking after baseline compared to the women who remained former or never smokers. No significant association with VPD emerged among the 351 women quitting smoking after baseline compared to the women who remained current smokers. Among the 648 women current smokers at baseline and follow-up a mean variation of -0.8 cigarettes smoked per day was observed (SD 6.1; min -23; max 52). No significant association with VPD emerged according to variation in number of cigarettes smoked per day among current smokers at baseline and at follow-up (**Table 4**). Likewise, no significant association emerged for the effect of changes in smoking habits between baseline and follow-up on DV (data not shown).

**Table 4:** Association between changes in smoking habits between baseline and follow up (mean 9.4 years) and volumetric percent mammographic density (VPD, %) among the 3,774 menopausal women from the EPIC Florence cohort.

	N (%)	Adjusted mean of VPD (95%CI) ^a^	Diff% ^b^	95%CI	p-value
Never or former smokers at baseline (n=2775)					
Never or former smokers at follow-up	2719 (98.0)	6.5 (6.4; 6.6)	Ref.		
Current smokers at follow-up	56 (2.0)	6.5 (5.9; 7.3)	-4.8	-14.6; 6.0	0.37
Current smokers at baseline (n=999)					
Current smokers at follow-up	648 (64.9)	6.2 (6.0; 6.5)	Ref.		
Former smokers at follow-up	351 (35.1)	6.6 (6.4; 6.8)	4.6	-1.2; 10.7	0.12
Current smokers at baseline and follow-up (n=648)					
Change in n. cigarette/day (quartiles):					
I (cigarette/day reduction: min -23; max -5)	147 (22,7)	6.6 (6.2; 7.1)	Ref.		
II (cigarette/day reduction: min -4; max -1)	239 (36.9)	6.4 (6.1; 6.8)	-7.3	-15.5; 1.7	0.11
III (cigarette/day increase: min 0; max +3)	126 (19.4)	7.2 (6.7; 7.8)	-0.1	-11.4; 12.7	0.99
IV (cigarette/day increase: min +4; max +52)	132 (20.4)	6.2 (5.8; 6.7)	-5.5	-15.1; 5.1	0.29
p trend					0.61

a Reported means and 95%CI are back-transformed from log-transformed estimated means from multivariable linear regression models.

b Multivariable linear regression models adjusted for age at menarche, age at menopause, birth index, BMI at follow up, waist circumference at follow up, RII, HRT at follow up, daily alcoholic units at follow up, age at mammographic examination, and mammographic breast thickness. The %Diff represents the percent change of VPD with respect to the reference category.

**Figure 2:**
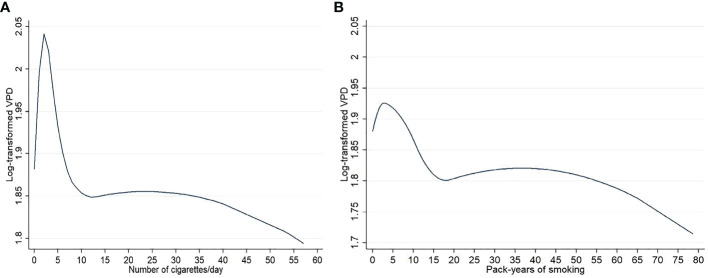
Graphs representing the dose effect of number of cigarettes/day **(A)** and pack-years of smoking **(B)** on log-transformed volumetric percent density (VPD) in the 3774 menopausal study women from the EPIC Florence cohort. Results from quadratic b spline analysis.

Results of the quadratic b-splines analyses for smoking intensity (number of cigarettes/day) and lifetime smoking exposure (pack-years), showed in **Figure 2**, substantially confirm the dose-response effect of smoking habits.

## Discussion

This prospective study carried out in a large series of menopausal women from the EPIC Florence study showed an inverse association between smoking exposure and VPD as measured after an average 5.3 years from smoking habits assessment, with an inverse dose-response relationship with numbers of cigarettes, years of smoking duration and pack-years smoked. These associations were similar, also if with an attenuated effect, when DV was used as the outcome variable.

Our finding of an inverse association between current active smoking and MBD supports the results of most previous studies among menopausal women despite the differences in study design (all previous studies had a cross-sectional design) and in method of MBD assessment, the latter spacing from categorical classifications (16, 24, 29) to computer-based assisted method (27, 28).

Two studies examined the association between smoking status and MBD assessed by volumetric Volpara™ density software. In 2018 Hjerkind et al. published a paper on MBD and BC risk factors in a large cohort of 45,448 women (50-69 years, 73.5% menopausal), who participated in the Norwegian screening program for BC between 2007 and 2014. In this cohort both percent and absolute MBD were slightly lower among current smokers (39). In 2021, Pepłońska et al. published a cross-sectional study on cigarette smoking and MBD in a series of 467 Polish women aged 40–60 years (79% menopausal) who underwent screening mammography in 2013–2018. The adjusted analyses showed an inverse significant association between the number of pack-years and volumetric MBD among the current smokers (40).

Few other studies found no association between smoking and MBD among postmenopausal women. In a cross-sectional study from Gapstur et al. published in 2003 and including 191 menopausal Hispanic women, no relationship emerged between smoking and mammographic density (41). In a previous cross-sectional study by Vachon et al. among American women participating in a large BC family study, no association between smoking and MBD emerged among the 1,554 menopausal women, while an inverse association was found among the 346 non-menopausal women (26).

The inverse association between smoking and MBD has a biological plausibility mostly attributable to the anti-estrogenic effect of smoking. It has been documented that tobacco smoke accelerates the metabolism of estradiol to metabolites with minimum estrogenic activity, inhibits aromatization of androgens into estrogens, and increases the binding of circulating estrogens by sex hormone-binding globulin. These effects together lead to a lower level of circulating estrogens and potentially to a decreased MBD (29, 42).

Our study has some limitations that deserve to be fully acknowledged. By being based on women who volunteered to join the EPIC study and the subsequent follow-up and also participated in the BC screening, the presence of health conscious people with healthy behaviors could be higher and the proportion of smokers could be lower than in the general population, possibly leading to lower estimates of the association with MBD measures. However, our data on the prevalence of smokers are quite similar to the data collected in the population of central Italy in the period in which the follow-up was carried out (43, 44).

Our study also has several strengths. First of all the FEDRA study is nested within a well-characterized general population-based cohort with plenty of information available on potential confounders of the association being studied. It included detailed information on active smoking that provided the possibility to examine multiple aspects of smoking exposure. It also included repeated information on smoking habits that provided the ability to examine the effect of the smoking habits changes on the MBD assessed some years later. The volumetric assessment of MBD from digital mammograms, through an automated and validated software, ensured the reproducibility of measures and the possibility of comparison with other published studies in the same field. The prospective design of our study, with MBD assessed after some years from the smoking exposure assessment, allowed us to confirm the cause-effect relation between smoking and MBD reduction that was observed in previous cross-sectional studies.

In conclusion, this longitudinal study confirms the inverse association between active smoking and MBD, probably due to the anti-estrogenic effects of tobacco smoking. However, cigarette smoking was recently defined as a risk factor for BC (5, 6), therefore suggesting that the causal biological pathway linking smoking and BC not includes MBD but is probably due to the carcinogenic effects of constituents in tobacco smoke on the breast tissue. Given the important anti-estrogenic effect on MBD and the established carcinogenic effect on breast tissue, it would be interesting to evaluate, in future studies, the effect of the inclusion of information on smoking habits during the lifespan as additional parameter in the main existing BC risk prediction models.

## Data availability statement

The raw data supporting the conclusions of this article are not publicly available due to participants privacy protection. They will be made available by the authors on reasonable request.

## Ethics statement

The studies involving humans were approved by Ethics Committee of “Azienda Sanitaria Firenze” and “Area Vasta Centro” of Tuscany Region. The studies were conducted in accordance with the local legislation and institutional requirements. The participants provided their written informed consent to participate in this study.

## Author contributions

BB: Formal Analysis, Visualization, Writing – original draft. SC: Formal Analysis, Methodology, Writing – review & editing. MA: Data curation, Formal Analysis, Writing – review & editing. IE: Investigation, Writing – review & editing. EP: Investigation, Writing – review & editing. LF: Data curation, Formal Analysis, Writing – review & editing. MG: Investigation, Writing – review & editing. GD: Formal Analysis, Writing – review & editing. MF: Investigation, Writing – review & editing. AQ: Data curation, Software, Writing – review & editing. DA: Investigation, Writing – review & editing. CS: Methodology, Supervision, Writing – review & editing. GM: Funding acquisition, Methodology, Supervision, Writing – original draft.
